# Oblique collision of ion acoustic solitons in a relativistic degenerate plasma

**DOI:** 10.1038/s41598-020-72449-x

**Published:** 2020-09-30

**Authors:** S. K. El-Labany, W. F. El-Taibany, E. E. Behery, Rami Abd-Elbaki

**Affiliations:** Department of Physics, Faculty of Science, Damietta University, P.O. Box 34517, New Damietta, Egypt

**Keywords:** Physics, Plasma physics

## Abstract

The interaction (oblique collision) of two ion acoustic solitons (IASs) in a magnetized relativistic degenerate plasma with relativistic degenerate electrons and non-degenerate cold ions is studied. The extended Poincaré–Lighthill–Kuo (PLK) method is used to obtain two Korteweg deVries (KdV) wave equations that describe the interacting IASs, then the phase shifts due to interaction are calculated. We studied influence of the fluid number density on the interaction process, interacting solitons phase shifts and also phase velocities. The introduced model is valid for astrophysical objects with high density matter such as white dwarfs, neutron stars, degenerate electrons gas in metals and laboratory degenerate plasma. An inverse proportionality between the phase shifts, phase velocity and the equilibrium electron fluid number density $n_{eo}$ was established in the range $10^{35}\,{\text {m}}^{-3}>n_{eo}>10^{38}\,{\text {m}}^{-3}$. We found that the soliton waves get sharper (narrower) and higher with increasing the electrons fluid number density $n_{eo}$, and hence less spacial occupying. The phase shifts and the phase velocity remain approximately unchanged in the range of $10^{35}\,{\text {m}}^{-3}<n_{eo}<10^{38}\,{\text {m}}^{-3}$. The impact of the obliqueness angle $\theta$ on the soliton interaction process is also studied.

## Introduction

The study of matter properties under extreme conditions has gained a growing interest of research^1–3^. Such excessive conditions occur in a number of astrophysical compact objects^4–7^, planetary systems and cosmic environments^7,8^. The neutron stars and white dwarfs are examples for such systems^1,9–11^. Higher density is a common property in the previously mentioned systems which causes degeneracy to form what is called a degenerate plasma where the mean interparticle separation $n^{-1/3}$ is comparable or smaller than the electrons de Broglie wavelength $\lambda _{B}$ i.e. $n\lambda _{B}^{3}\ge 1$; $\lambda _{B}=\frac{h}{\left( 2\pi m_{e}k_{B}T\right)^{1/2}}$^12,13^, where n stands for the particle number density. Under such conditions the relativistic and quantum impacts are unavoidable and the relativistic degeneracy pressure which arises as a result of the Pauli-Exclusion mechanism must be taken in to consideration. The high degenerate matter density in such compact objects, which are considered as “relics of stars” that have reached the end of burning thermonuclear fuel, and as a consequence no production of thermal pressure anymore. Lack of thermal pressure results in a size shrinking significantly, which in turn makes the density of their interiors to become extremely high. Under this situation, these objects generate nonthermal pressure via fermion (electron) degenerate pressure and also particle-particle interactions.

The observational evidences besides theoretical analysis indicate that these compact objects, which support themselves against the gravitational collapse through cold fermion (electron) degenerate pressure, are of two categories. The white dwarf is one of the first category examples which is supported by the pressure of degenerate electrons whose interior is not far from being a dense solid ion lattice that is surrounded by degenerate electrons. The neutron star on the other hand is classified as an example of the second category whose interior is near to a giant atomic nucleus that is a mixture of interacting electrons, nucleons and may be other elementary particles which is supported by the pressure due to a combination of nuclear interactions besides nucleon degeneracy. Such unique states and extreme matter conditions occur due to the significant compression of the interstellar medium^14,15^, where the electron degenerate pressure doesn’t depend on the electron temperature but only relies on the electron number density. The astrophysical objects mentioned earlier have a very high density, for example, the degenerate electron number density in a typical white dwarf is in the order of $10^{30}\;{\text {cm}}^{-3}$ or even more^14,16^ and hence the Fermi energy for the electrons is comparable to its mass energy and therefore the electron speed is in comparison to the light speed in vacuum. These compact interstellar objects provide us cosmic laboratories for analyzing the medium matter properties as well as waves and instabilities^17–20^ at an excessive high-density degenerate situation in such a medium for which quantum besides relativistic effects become important^17,21^.

Scientists gave a great interest to the study and analysis of the nonlinear dynamics of Ion Acoustic Solitons (IASs) in magnetized plasmas^22–25^ and a special interest for the unstable relativistic and ultrarelativistic degeneracy state^26–29^. The importance of such a field is to understand the role of degeneracy when combined with relativistic or ultrarelativistic speeds. For example the electron degeneracy in the massive white dwarfs which holds it against their gravitational pressure gets soften as the electrons obtain relativistic leading to further gravitational star collapse^30^. An increase of the steepness and strength of a quantum ion acoustic shock wave with the decrease of the stretched time coordinates was founded by Masood *et al.*^31^. The quantum corrections and raising positron concentration impact on the phase shifts was examined by El-Labany *et al.* in a dense quantum plasma composed of electron-positron-ion^32^. El-Taibany and Mamun have studied electromagnetic perturbations in electron-positron degenerate ultrarelativistic plasma^15^. Zobaer *et al.* studied electrostatic shock structures and their fundamental features in a degenerate dense plasma that contains both nonrelativistic and ultrarelativistic degenerate electrons and cold non- relativistic degenerate ions^33^. Behery *et al.* studied the propagation and stability of non-linear solitons by getting the Zakharov-Kuznetsov equation in a supersonic relativistic quantum plasma indicating their despersion properties and highlighting the possible applications in both space and laboratory plasma^34^. Choudhury *et al.*^35^ have studied a two soliton interaction in a semiconductor of quantum plasma, also they investigated the effect of quantum diffraction parameter and hole to electron equilibrium density ratio on the phase shifts.

The oblique interaction in solitons occurs when the two solitons approach each other at an obliqueness angle $\theta$ where $0<\theta <180$. Akbari-Moghanjoughi *et al*^36,37^ studied the electron acoustic solitons that obliquely interact in two electron populated quantum degenerate plasmas where they discussed critical quantum diffraction parameter and its effect on solitons’ types and their interaction phase shifts. Moreover they considered the collision angle and fractional plasma constituents concentration. The influence of the positron concentration, electron superthermality and obliqueness of magnetic field on the soliton-cnoidal wave are investigated by^38^ in detail. The plasma parameters that exist in white dwarf stars for the fast and slow modes of magnetoacoustic waves are used by^39^ to study the interaction of obliquely propagating solitons. Iqbal Shaukat^40^ studied the impact of the quantizing magnetic field inclusion on the solitary wave propagation characteristics which may be of interest in understanding the nonlinear electrostatic structures propagation in dense astrophysical environments such as white dwarfs. The particle-in-cell simulations using both one-dimensional and two-dimensional was used by Wu *et al*^41^ and the formation besides basic properties of these long-lived electromagnetic relativistic solitons are studied. Theoretical and experimental observation of multi-soliton formation in femtosecond degenerate optical parametric oscillators (OPOs) by Ning and Zhang^42^. Cole *et al*^43^ reported the observation of soliton crystals in monolithic Kerr microresonators–spontaneously and collectively ordered ensembles of co-propagating solitons whose interactions discretize their allowed temporal separations. There are collective nonlinear wave–wave interactions in dense plasmas like intense laser–solid density plasma experiments^44,45^, astrophysical superdense bodies (e.g. the white dwarf core and neutron stars)^46,47^ and the micro and nano scale quantum diodes^48–50^.

It is of interest to propose 3D plasma model that allows both the degeneracy and relativistic features and can be applicable to the previously mentioned plasmas as in laser–solid density plasma experiments, astrophysical superdense bodies and the micro and nano scale physical entities like quantum diodes . Specifically, in this work we studied the ion-acoustic excitations in an electrostatic plasma model, where the electrons are considered to be degenerate and on the other hand, a cold nondegenerate ion fluid due to their larger mass is considered. In this paper we introduce the propagation and interaction of nonlinear pulses named solitons under the effect of fluid number density. Our main aspect that is studied in this research is the impact of changing the fluid number densities on the solitary pulses including their interaction (oblique collision), phase velocities and phase shifts. This paper will be organized as follows: in the hydrodynamic model section we introduce the basic set of normalized equations describing our plasma model. In the nonlinear analysis section a nonlinear analysis for our system is done with the help of suitable asymptotic expansion to solve our plasma system which is the Poincaré–Lighthill–Kuo (PLK) method^51,52^ to get two Korteweg deVries (KdV) wave equations that describe the interacting IASs and also the phase shifts due to interaction are calculated. All the numerical analysis and discussion are presented in the section that comes after. Finally there is a conclusion section that summarizes our work.

## The hydrodynamic model

In the existence of a static external magnetic field $B=B_{o}{\hat{z}}$ along z direction with the unit vector ${\hat{z}}$ such that $B_{o}$ is the magnetic field strength, considering the movement of ion acoustic (IA) excitations in a relativistic degenerate plasma. We consider a two component degenerate relativistic plasma composed of ions and electrons. Introducing the normalized set of governing equations that were adopted from^34^ as follows:

1 $$\begin{aligned}&\frac{\partial }{\partial t}\left( \gamma _{i}n_{i}\right) +\nabla \cdot \left( \gamma _{i}n_{i}\mathbf {u_{i}}\right) =0, \end{aligned}$$

2 $$\begin{aligned}&\frac{\partial }{\partial t}\left( \gamma _{e}n_{e}\right) +\nabla \cdot \left( \gamma _{e}n_{e}\mathbf {u_{e}}\right) =0, \end{aligned}$$

3 $$\begin{aligned}&\frac{\partial }{\partial t}\left( \gamma _{i}\mathbf {u_{i}}\right) +\left( \mathbf {u_{i}}\cdot \nabla \right) \left( \gamma _{i} \mathbf {u_{i}}\right) +\nabla \phi -\Omega \left( \mathbf {u_{i}}\times {\hat{\mathbf {z}}}\right) =0, \end{aligned}$$

4 $$\begin{aligned}&\nabla \phi -\Omega \left( \mathbf {u_{e}}\times {\hat{\mathbf {z}}}\right) -\frac{\beta \gamma _{e}\alpha _{o}^{2}n_{e}^{-1/3}}{\left( 1+\alpha _{o}^{2}n_{e}^{2/3}\right) } \left[ \nabla +\delta \mathbf {u_{e}}\frac{\partial }{\partial t}\right] n_{e}=0, \end{aligned}$$

5 $$\begin{aligned}&\nabla ^{2}\phi -\left( \gamma _{e}n_{e}-\gamma _{i}n_{i}\right) =0. \end{aligned}$$

This system has been declared with $n_{i}$ and $n_{e}$ represent the fluid number density for ions and electrons, respectively, $\mathbf {u_{i}}$ and $\mathbf {u_{e}}$ are their fluids velocity. $m_{e}$ and *e* denote the electron mass and charge respectively, $m_{i}$ is the ion mass, *c* is the speed of light and $\gamma _{j}=\left( 1-c^{-2}(V_o\mathbf {u_j})^2 \right) ^{-\frac{1}{2}}$ where *j* is *i* for ions and *e* for electrons and $V_o=\left( \frac{E_{F_{e}}}{m_{i}}\right) ^{{1}/{2}}$. Also $\Omega =\frac{ez_{i}B_{o}}{m_{i}\omega _{pi}}=\frac{\omega _{ci}}{\omega _{pi}}$, $\beta =\frac{m_{e}c^{2}z_i}{3E_{Fe}}$ and $\delta =\frac{E_{Fe}}{m_ic^{2}}$, such that $+z_{i}e$ is the ionic charge and $E_{Fe}=\sqrt{P_{Fe}^{2}c^{2}+m_{e}^{2}c^{2}}-m_{e}c^{2}$ is the relativistic Fermi energy for electrons. We have done the normalization such that *t*, $\nabla$, $\mathbf {u_e}$, $\mathbf {u_i}$ and $\phi$ were normalized by $\omega _{pi}^{-1}=\left( \frac{\in _{o}m_{i}}{e^{2}z_{i}^{2}n_{io}}\right) ^{{1}/{2}}$, $\left( \frac{\in _{o}E_{F_{e}}}{e^{2}z_{i}^{2}n_{io}}\right) ^{{-1}/{2}}$, $\left( \frac{E_{F_{e}}}{m_{i}}\right) ^{{1}/{2}}$, $\left( \frac{E_{F_{e}}}{m_{i}}\right) ^{{1}/{2}}$ and $\frac{E_{F_{e}}}{ez_{i}}$ respectively.

Introducing the parameter $\alpha$ as:

6 $$\begin{aligned} \alpha =\frac{P_{Fe}}{m_{e}c^{2}}=\frac{\hbar }{m_{e}c}\left( 3\pi ^{2} n_{e}\right) ^{{1}/{3}}, \end{aligned}$$

where $P_{Fe}$ is the electron Fermi momentum. The electron pressure ${{\mathscr {P}}}_{e}$ is given by:

7 $$\begin{aligned} {{\mathscr {P}}}_{e}=\frac{m_{e}^{4}c^{5}}{24\pi ^{2}\hbar ^{3}} \left[ \alpha \left( 2\alpha ^{2}-3\right) \left( \alpha ^{2}+1 \right) ^{{1}/{2}}+3\sinh ^{-1}\alpha \right] , \end{aligned}$$

defining $\rho _{e}$ as the internal energy density of the electron fluid which is related to to ${{\mathscr {P}}}_e$ through the relation:

8 $$\begin{aligned} {{\mathscr {P}}}_{e}+\rho _{e}=n_{e}m_{e}c^{2}\sqrt{\alpha ^{2}+1}. \end{aligned}$$

Presuming $n_{eo}$ is the equilibrium electron fluid number density, so $n_{eo}=z_{i}n_{io}$ is the condition for charge neutrality at equilibrium for the proposed model. As for the equilibrium state, we have

$$\begin{aligned} \alpha =\alpha _{o}\left( \frac{n_{e}}{n_{eo}}\right) ^{{1}/{3}} \qquad \text {with}\qquad \alpha _{o}=\frac{\hbar }{m_{e}c}\left( 3\pi ^{2} n_{eo}\right) ^{{1}/{3}} \end{aligned}$$

The Fermi and Bohm pressure terms contribute to the electron pressure, however the Bohm term will be omitted in this study because it dominates at very small wavelengths near to or smaller than the inter particle mean distance which causes a break down for the fluid model at extremely small wavelengths^53^.

## Oblique collision

To study the oblique collision of two ion acoustic (IA) excitations, namely solitons (as comes latter), Poincaré–Lighthill–Kuo (PLK) method will be employed to investigate the collision process of these two solitons traveling in two arbitrary directions at an angle $\theta :0<\theta <180$. Now we assume that the two solitons denoted as $S_{1}$ and $S_{2}$ in an initial state such that the two solitons are asymptotically far apart and travel obliquely toward each other. After some time, an interaction, a collision occurs and then departure. We also suppose that the two solitons interact with each other weakly. Hence, the collision is expected to be quasi elastic. According to the PLK method, we expand the dependent variables as

9 $$\begin{aligned} \begin{aligned} n_{j}&=1+\epsilon ^{2}n_{j1}+\epsilon ^{3}n_{j2}+\epsilon ^{4}n_{j3}+\cdots ,\\ u_{jx}&=\epsilon ^{3}u_{jx1}+\epsilon ^{4}u_{jx2}+\epsilon ^{5}u_{jx3}+\cdots ,\\ u_{jy}&=\epsilon ^{3}u_{jy1}+\epsilon ^{4}u_{jy2}+\epsilon ^{5}u_{jy3}+\cdots ,\\ u_{jz}&=\epsilon ^{2}u_{jz1}+\epsilon ^{3}u_{jz2}+\epsilon ^{4}u_{jz3}+\cdots ,\\ \phi&=\epsilon ^{2}\phi _{1}+\epsilon ^{3}\phi _{2}+\epsilon ^{4}\phi _{3}+\cdots , \end{aligned} \end{aligned}$$

where *j* will stand for ions as *i* and electrons as *e*. The non linearity strength is characterized by $\epsilon$ which is a small parameter. Introducing $\xi$ and $\eta$ as the trajectories (independent variables) of the two solitons $S_1$ and $S_2$ which are given as the following stretched coordinates.

10 $$\begin{aligned} \begin{aligned} \xi&=\epsilon \left( l_{x}x+l_{y}y+l_{z}z-\lambda t\right) + \epsilon ^{2}P_{o}(\eta ,\tau )+\epsilon ^{3}P_{1}(\eta ,\tau )+\cdots ,\\ \eta&=\epsilon \left( l_{x}^{'}x+l_{y}^{'}y+l_{z}^{'}z+\lambda ^{'}t \right) +\epsilon ^{2}Q_{o}(\xi ,\tau )+\epsilon ^{3}Q_{1}(\xi ,\tau )+\cdots ,\\ \tau&=\epsilon ^{3}t, \end{aligned} \end{aligned}$$

where $\lambda$ and $\lambda ^{'}$ are the phase velocities of the IA soliton waves. $S_{1}$ and $S_{2}$ move in two different directions with $R_{1}=l_{x}x+l_{y}y+l_{z}z$ and $R_{2}=l_{x}^{'}x+l_{y}^{'}y+l_{z}^{'}z$, respectively, such that $\left( l_{x},l_{y},l_{z}\right)$ and $(l_{x}^{'},l_{y}^{'},l_{z}^{'})$ represent the directional cosines of $S_1$ and $S_2$ wave vector along the *x*, *y* and *z* axes, respectively. $P_{o}(\eta ,\tau )=Q_{o}(\xi ,\tau )=0$ at the initial state of the two solitons $S_{1}$ and $S_{2}$ but after the interaction, a change in the solitons’ trajectories takes place and hence $P_{o}(\eta ,\tau )\ne 0\quad \text {and}\quad Q_{o}(\xi ,\tau )\ne 0$ and to be determined later. The angle between the propagation directions of the two solitons is given explicitly by $\theta =\cos ^{-1}\{{(l_{x}l_{x}^{'}+l_{y}l_{y}^{'}+l_{z}l_{z}^{'})}/{[(l_{x}^{2}+l_{y}^{2}+l_{z}^{2})^{{1}/{2}}(l_{x}^{'2}+l_{y}^{'2}+l_{z}^{'2})^{{1}/{2}} ] } \}$^54^.

After transforming from *x*, *y* and *z* space to $\xi , \eta$ and $\tau$ space, now we can substitute Eqs. (9) and (10) into Eqs. (1) – (5). Using the following solvability condition

11 $$\begin{aligned} \phi {}_{1}(\xi ,\eta ,\tau )=\phi {}_{1}^{\xi }(\xi ,\tau )+ \phi {}_{1}^{\eta }(\eta ,\tau ), \end{aligned}$$

then collecting the terms of the same $\epsilon$ powers; we get for the lowest $\epsilon$ order:

12 $$\begin{aligned} n_{e1}&=\frac{\left( 1+\alpha _{o}^{2}\right) }{\beta \alpha _{o}^{2}} \left( \phi {}_{1}^{\xi }+\phi {}_{1}^{\eta }\right) \\ n_{i1}&=\frac{l_{z}^{2}}{\lambda ^{2}}\phi {}_{1}^{\xi }+\frac{l_{z}^{'2}}{\lambda ^{'^{2}}}\phi {}_{1}^{\eta } \end{aligned},$$

13 $$\begin{aligned}u_{ez1}&=\frac{\left( 1+\alpha _{o}^{2}\right) }{\beta \alpha _{o}^{2}} \left( \frac{\lambda }{l_{z}}\phi {}_{1}^{\xi }-\frac{\lambda ^{'}}{l_{z}^{'}} \phi {}_{1}^{\eta }\right) \\ u_{iz1}&\frac{l_{z}}{\lambda }\phi {}_{1}^{\xi }-\frac{l_{z}^{'}}{\lambda ^{'}}\phi {}_{1}^{\eta } \end{aligned},$$

14 $$\begin{aligned}u_{ix1}&=-\frac{1}{\Omega }\left( l_{y}\frac{\partial }{\partial \xi } \phi {}_{1}^{\xi }+l_{y}^{'}\frac{\partial }{\partial \eta }\phi {}_{1}^{\eta }\right) \\ u_{iy1}&=\frac{1}{\Omega }\left( l_{x}\frac{\partial }{\partial \xi } \phi {}_{1}^{\xi }+l_{x}^{'}\frac{\partial }{\partial \eta }\phi {}_{1}^{\eta }\right) \end{aligned},$$

15 $$\begin{aligned}&u_{ex1}=u_{ey1}=0 . \end{aligned}$$

The set of Eqs. (12) – (14) are used to obtain the phase velocities $\lambda$ and $\lambda ^{'}$ as

16 $$\begin{aligned} \begin{array}{ccc} \lambda =l_{z}\alpha _{o}\sqrt{\frac{\beta }{\left( 1+\alpha _{o}^{2}\right) }}&\text {and}&\lambda ^{'}=l_{z}^{'}\alpha _{o}\sqrt{\frac{\beta }{\left( 1+\alpha _{o}^{2}\right) }} \end{array}, \end{aligned}$$

as a consequence, it leads to $n_{i1}=n_{e1}$ and $u_{iz1}=u_{ez1}$ from Eqs. (12), (13) and (16).

For the next higher $\epsilon$ order, we have

17 $$\begin{aligned} \phi {}_{2}(\xi ,\eta ,\tau )=\phi {}_{2}^{\xi }(\xi ,\tau )+\phi {}_{2}^{\eta } (\eta ,\tau ). \end{aligned}$$

Thus, we get the values of the next order of perturbed dependent variables as

18 $$\begin{aligned}n_{e2}&=\frac{\left( 1+\alpha _{o}^{2}\right) }{\beta \alpha _{o}^{2}} \left( \phi {}_{2}^{\xi }+\phi {}_{2}^{\eta }\right) \\ n_{i2}&=\frac{l_{z}^{2}}{\lambda ^{2}}\phi {}_{2}^{\xi }+\frac{l_{z}^{'2}}{\lambda ^{'2}}\phi {}_{2}^{\eta } \end{aligned},$$

19 $$\begin{aligned}u_{ez2}&=\frac{\left( 1+\alpha _{o}^{2}\right) }{\beta \alpha _{o}^{2}} \left( \frac{\lambda }{l_{z}}\phi {}_{2}^{\xi }-\frac{\lambda ^{'}}{l_{z}^{'}} \phi {}_{2}^{\eta }\right) \\ u_{iz2}&=\left( \frac{l_{z}}{\lambda }\phi {}_{2}^{\xi }-\frac{l_{z}^{'}}{\lambda ^{'}}\phi {}_{2}^{\eta }\right) \end{aligned},$$

20 $$\begin{aligned}u_{ix2}&=\frac{1}{\Omega }\left( \frac{\lambda l_{x}}{\Omega } \frac{\partial ^{2}}{\partial \xi ^{2}}\phi _{1}^{\xi }- \frac{\lambda ^{'}l_{x}^{'}}{\Omega }\frac{\partial ^{2}}{\partial \eta ^{2}} \phi _{1}^{\eta }-l_{y}\frac{\partial }{\partial \xi }\phi {}_{2}^{\xi }- l_{y}^{'}\frac{\partial }{\partial \eta }\phi {}_{2}^{\eta }\right) \\ u_{iy2}&=\frac{1}{\Omega }\left( \frac{\lambda l_{y}}{\Omega } \frac{\partial ^{2}}{\partial \xi ^{2}}\phi _{1}^{\xi }- \frac{\lambda ^{'}l_{y}^{'}}{\Omega }\frac{\partial ^{2}}{\partial \eta ^{2}} \phi _{1}^{\eta }+l_{x}\frac{\partial }{\partial \xi }\phi {}_{2}^{\xi }+ l_{x}^{'}\frac{\partial }{\partial \eta }\phi {}_{2}^{\eta }\right) \end{aligned},$$

21 $$\begin{aligned}&u_{ex2}=u_{ey2}=0. \end{aligned}$$

Also as a result from the consistence of the above relations, we have $u_{iz2}=u_{ez2}$. For the next higher order of $\epsilon$, we get the following equation

22 $$ \begin{aligned} - 2{\text{(}}\lambda l^{\prime}_{z} + \lambda ^{\prime}l_{z} {\text{)}}u_{{iz3}} = & 2\frac{{l_{z}^{2} }}{\lambda }\int {\left( {\frac{{\partial \phi _{1}^{\xi } }}{{\partial \tau }} + A_{1} \phi _{1}^{\xi } \frac{{\partial \phi _{1}^{\xi } }}{{\partial \xi }} + B_{1} \frac{{\partial ^{3} \phi _{1}^{\xi } }}{{\partial \xi ^{3} }}} \right)} d\eta \\   &+ 2l^{\prime 2}_{z} \lambda \int {\left( {\frac{{\partial \phi _{1}^{\eta } }}{{\partial \tau }} - A_{2} \phi _{1}^{\eta } \frac{{\partial \phi _{1}^{\eta } }}{{\partial \eta }} - B_{2} \frac{{\partial ^{3} \phi _{1}^{\eta } }}{{\partial \eta ^{3} }}} \right)} d\xi \\   &- \iint {\left( {D\frac{{\partial P_{o} }}{{\partial \eta }} - E_{1} \phi _{1}^{\eta } } \right)}\frac{{\partial ^{2} \phi _{1}^{\xi } }}{{\partial \xi ^{2} }}d\xi d\eta \\  & + \iint {\left( {D\frac{{\partial Q_{o} }}{{\partial \xi }} - E_{2} \phi _{1}^{\xi } } \right)}\frac{{\partial ^{2} \phi _{1}^{\eta } }}{{\partial \eta ^{2} }}d\xi d\eta \\ \end{aligned} $$

where

23 $$\begin{aligned} A_{1}=&3\frac{l_{z}^{2}}{\lambda }-2\lambda -\frac{\delta \lambda }{2}- \frac{\left( 1+3\alpha _{o}^{2}\right) \lambda }{3\beta \alpha _{o}^{2}}, \end{aligned}$$

24 $$\begin{aligned} A_{2}=&3\frac{l_{z}^{'2}}{\lambda ^{'}}-2\lambda ^{'}-\frac{\delta \lambda ^{'}}{2}- \frac{\left( 1+3\alpha _{o}^{2}\right) \lambda ^{'}}{3\beta \alpha _{o}^{2}}, \end{aligned}$$

25 $$\begin{aligned} B_{1}=&\frac{\lambda ^{3}}{2l_{z}^{2}}\left[ \left( l_{x}^{2}+l_{y}^{2}+ l_{z}^{2}\right) +\frac{1}{\Omega ^{2}}\left( l_{x}^{2}+l_{y}^{2}\right) \right] , \end{aligned}$$

26 $$\begin{aligned} B_{2}=&\frac{\lambda ^{'^{3}}}{2l_{z}^{'2}}\left[ \left( l_{x}^{'2}+l_{y}^{'2} +l_{z}^{'2}\right) +\frac{1}{\Omega ^{2}}\left( l_{x}^{'2}+l_{y}^{'2}\right) \right] , \end{aligned}$$

27 $$\begin{aligned} E_{1}=&\frac{\delta l_{z}^{2}}{2}-\frac{l_{z}^{4}}{\lambda ^{2}}- \frac{\left( 1+3\alpha _{o}^{2}\right) }{3\beta \alpha _{o}^{2}} l_{z}^{2}-l_{z}^{2}, \end{aligned}$$

28 $$\begin{aligned} E_{2}=&\frac{\delta l_{z}^{'2}}{2}-\frac{l_{z}^{'4}}{\lambda ^{'2}} -\frac{\left( 1+3\alpha _{o}^{2}\right) }{3\beta \alpha _{o}^{2}}l_{z}^{'2} -l_{z}^{'2}, \end{aligned}$$

29 $$\begin{aligned} D=&\, -4l_{z}l_{z}^{'} \end{aligned}$$

and considering the right hand side of Eq. (22), the proportionality to $\eta \left( \xi \right)$ of the first (second) term requires that these two terms to be secular terms as a reason for the independence of the integrated functions on $\eta \left( \xi \right)$. To avoid spurious resonances, we must eliminate those secular terms, so we have

30 $$\begin{aligned}&\frac{\partial \phi {}_{1}^{\xi }}{\partial \tau }+A_{1}\phi _{1}^{\xi } \frac{\partial \phi {}_{1}^{\xi }}{\partial \zeta }+B_{1}\frac{\partial ^{3} \phi {}_{1}^{\xi }}{\partial \zeta ^{3}}=0, \end{aligned}$$

31 $$\begin{aligned}&\frac{\partial \phi {}_{1}^{\eta }}{\partial \tau }-A_{2}\phi _{1}^{\eta } \frac{\partial \phi {}_{1}^{\eta }}{\partial \eta }-B_{2}\frac{\partial ^{3} \phi {}_{1}^{\eta }}{\partial \eta ^{3}}=0. \end{aligned}$$

Returning again to Eq. (22), for this order of $\epsilon$, The third and fourth terms are not secular terms. However, for the next order they will be^55,56^. Hence, we get the following equations for the leading phase shifts as

32 $$\begin{aligned} D\frac{\partial P_{o}}{\partial \eta }=E_{1}\phi _{1}^{\eta }, \end{aligned}$$

and

33 $$\begin{aligned} D\frac{\partial Q_{o}}{\partial \zeta }=E_{2}\phi _{1}^{\xi }. \end{aligned}$$

Equations (30) and (31) represent two-side traveling KdV wave equations in $\xi$ and $\eta$ reference frames, respectively and they lead to the corresponding IA solitary wave solutions as

34 $$\begin{aligned} \phi {}_{1}^{\xi }= & {} \phi {}_{1m}^{\xi }\text {sech}^{2} \left[ \left( \frac{A_{1}\phi {}_{1m}^{\xi }}{12B_{1}}\right) ^{\frac{1}{2}} \left( \xi -\frac{A_{1}\phi {}_{1m}^{\xi }}{3}\tau \right) \right] , \end{aligned}$$

35 $$\begin{aligned} \phi {}_{1}^{\eta }= & {} \phi {}_{1m}^{\eta }\text {sech}^{2} \left[ \left( \frac{A_{2}\phi {}_{1m}^{\eta }}{12B_{2}}\right) ^{\frac{1}{2}} \left( \eta +\frac{A_{2}\phi {}_{1m}^{\eta }}{3}\tau \right) \right] , \end{aligned}$$

where $\phi {}_{1m}^{\xi }$ and $\phi {}_{1m}^{\eta }$ are the amplitudes of the two solitons $S_{1}$ and $S_{2}$, respectively in their initial position. Due to the collision, leading phase changes occur which can be calculated using Eqs. (32) and (33) to get

36 $$\begin{aligned} P_{o}\left( \eta ,\tau \right)= & {} \frac{E_{1}}{D} \left( \frac{12B_2\phi {}_{1m}^{\eta }}{A_2}\right) ^{\frac{1}{2}}\times \left\{ \tanh \left[ \left( \frac{A_{2}\phi {}_{1m}^{\eta }}{12B_{2}} \right) ^{\frac{1}{2}}\left( \eta +\frac{A_{2}\phi {}_{1m}^{\eta }}{3} \tau \right) \right] +1\right\} , \end{aligned}$$

37 $$\begin{aligned} Q_{o}\left( \xi ,\tau \right)= & {} \frac{E_{2}}{D}\left( \frac{12B_{1} \phi {}_{1m}^{\xi }}{A_{1}}\right) ^{\frac{1}{2}}\times \left\{ \tanh \left[ \left( \frac{A_{1}\phi {}_{1m}^{\xi }}{12B_{1}} \right) ^{\frac{1}{2}}\left( \xi -\frac{E_{1}\phi {}_{1m}^{\xi }}{3} \tau \right) \right] -1\right\} . \end{aligned}$$

For an oblique collision, the trajectories $\xi$ and $\eta$ of the two solitary waves can be reformed using Eqs. (36) and (37) to be

38 $$\begin{aligned} \xi= & {} \epsilon \left( l_{x}x+l_{y}y+l_{z}z-\lambda t\right) + \epsilon ^{2}\frac{E_{1}}{D}\left( \frac{12B_{2}\phi {}_{1m}^{\eta }}{A_{2}} \right) ^{\frac{1}{2}} \nonumber \\&\times \left\{ \tanh \left[ \left( \frac{A_{2}\phi {}_{1m}^{\eta }}{12B_{2}} \right) ^{\frac{1}{2}}\left( \eta +\frac{A_{2}\phi {}_{1m}^{\eta }}{3} \tau \right) \right] +1\right\} +\cdots , \end{aligned}$$

39 $$\begin{aligned} \eta= & {} \epsilon \left( l_{x}^{'}x+l_{y}^{'}y+l_{z}^{'}z+ \lambda ^{'}t\right) +\epsilon ^{2}\frac{E_{2}}{D}\left( \frac{12B_{1} \phi {}_{1m}^{\xi }}{A_{1}}\right) ^{\frac{1}{2}} \nonumber \\&\times \left\{ \tanh \left[ \left( \frac{A_{1}\phi {}_{1m}^{\xi }}{12B_{1}} \right) ^{\frac{1}{2}}\left( \xi -\frac{A_{1}\phi {}_{1m}^{\xi }}{3} \tau \right) \right] -1\right\} +\cdots . \end{aligned}$$

In order to get the phase shifts resulting from the oblique collision process, we suppose that the two solitons $S_{1}$ and $S_{2}$ are at the initial time ($t=-\infty$) asymptotically far from each other such that soliton $S_{1}$ is at $\xi =0\;\text {and}\;\eta =-\infty$ while soliton $S_{2}$ is at $\eta =0\;\text {and}\;\xi =+\infty$. After the oblique collision occurs i.e. at ($t=+\infty$), the soliton $S_{2}$ is at $\eta =0\;\text {and}\;\xi =-\infty$ far to the left of soliton $S_{1}$ which is at $\xi =0\;\text {and}\;\eta =+\infty$. Defining $\triangle P_{o}$ and $\triangle Q_{o}$ to be the corresponding phase shifts which can be estimated as follows^54,56^

40 $$\begin{aligned} \triangle P_{o}=-2\epsilon ^{2}\frac{E_{1}}{D} \left( \frac{12B_{2}\phi {}_{1m}^{\eta }}{A_{2}}\right) ^{\frac{1}{2}} \end{aligned}$$

and

41 $$\begin{aligned} \triangle Q_{o}=+2\epsilon ^{2}\frac{E_{2}}{D}\left( \frac{12B_{1} \phi {}_{1m}^{\xi }}{A_{1}}\right) ^{\frac{1}{2}}. \end{aligned}$$

Equations (40) and (41) indicate that a negative phase shift for each soliton in its propagation direction occurs as the soliton $S_{1}$ is traveling to the right while the soliton $S_{2}$ is traveling to the left. The negative phase shifts implies that the trajectories of the propagated solitons have a lagging behind the expected if they just leaved each other with no interaction^56,57^.

## Numerical analysis and discussions

The oblique collision process of a relativistic degenerate two species plasma containing ions and electrons is studied in this paper under the effect of many important parameters that influence the interaction procedure. Our main interest is to study the impact of fluid number densities on the interaction process. The two derived KdV equations for the two solitons will be studied numerically under the effect of interaction. We also numerically investigate the phase shifts $\triangle P_{o}$ and $\triangle Q_{o}$. We expressed all the vales in SI unit system, so physical quantities like *e*, $\hbar$, $m_{e}$, $\epsilon _{o}$ and *c* are all having their SI value while other parameters, let us use the following numerical value $l_{z}=0.1-0.9$, $l_{z}^{'}=-l_{z}$, $l_{y}=l_{y}^{'}=l_{x}^{'}=l_{x}$, $z_{i}=1$, and finally the smallness parameter $\epsilon =0.01$.

**Figure 1:**
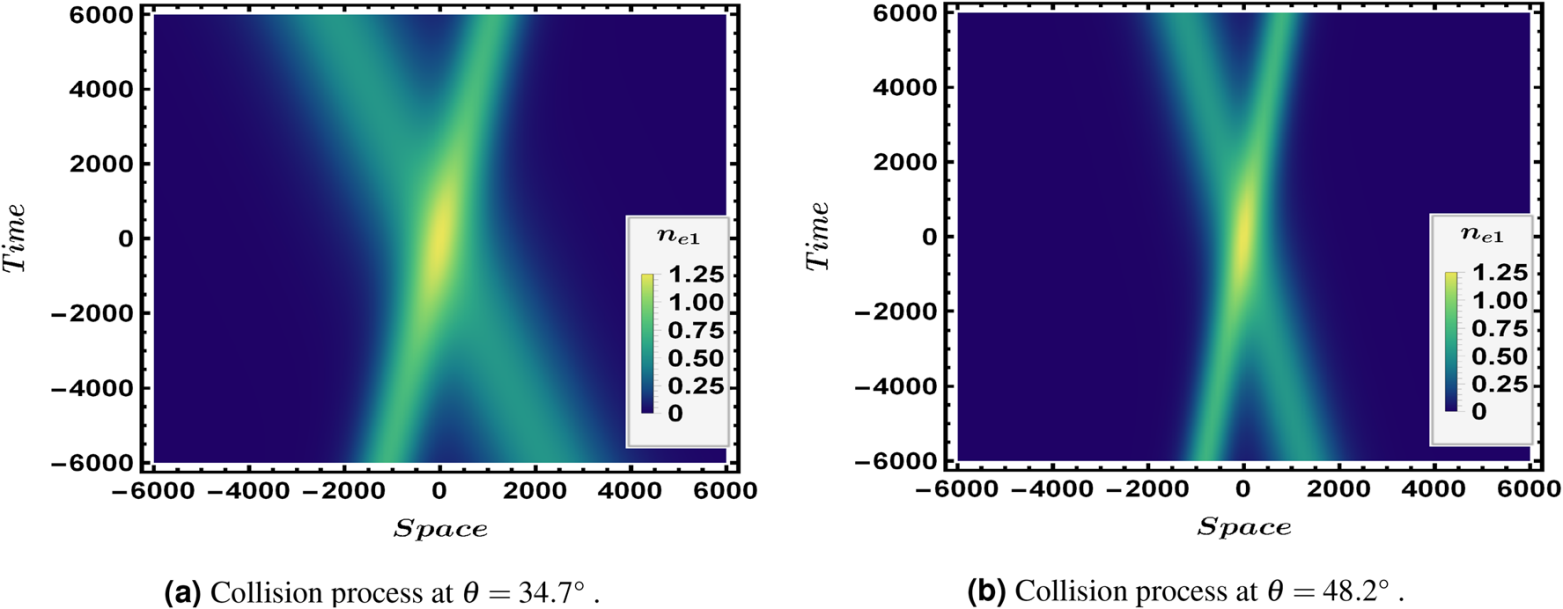
An overview, in a density profile, for the two solitons interaction at different values of obliqueness angle $$\theta $$ with , $$\epsilon =0.01$$, $$n_{eo}=10^{35} $$, $$\phi {}_{1m}^{\eta }=0.3$$, $$\phi {}_{1m}^{\xi }=0.4$$ and $$\Omega =0.2$$.

**Figure 2:**
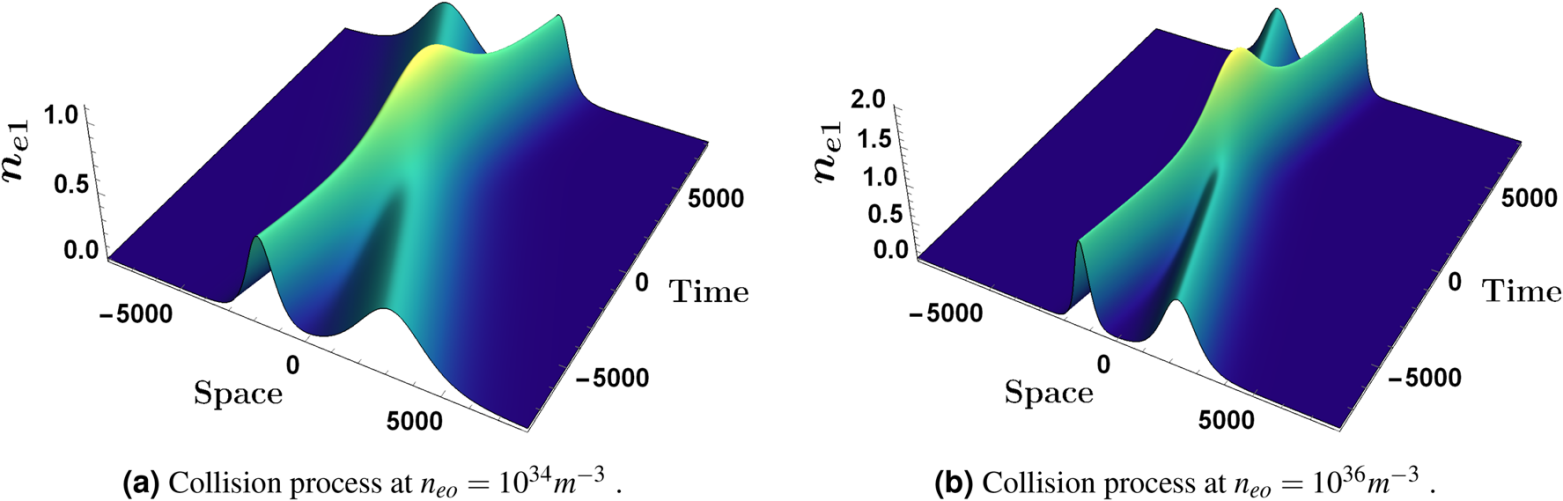
An overview, in a 3-D profile, for the two solitons interaction at different values of $n_{eo}$ with , $\epsilon =0.01$, $\theta =48.2 {^{\circ }}$, $\phi {}_{1m}^{\eta }=0.3$, $\phi {}_{1m}^{\xi }=0.4$ and $\Omega =0.2$.

**Figure 3:**
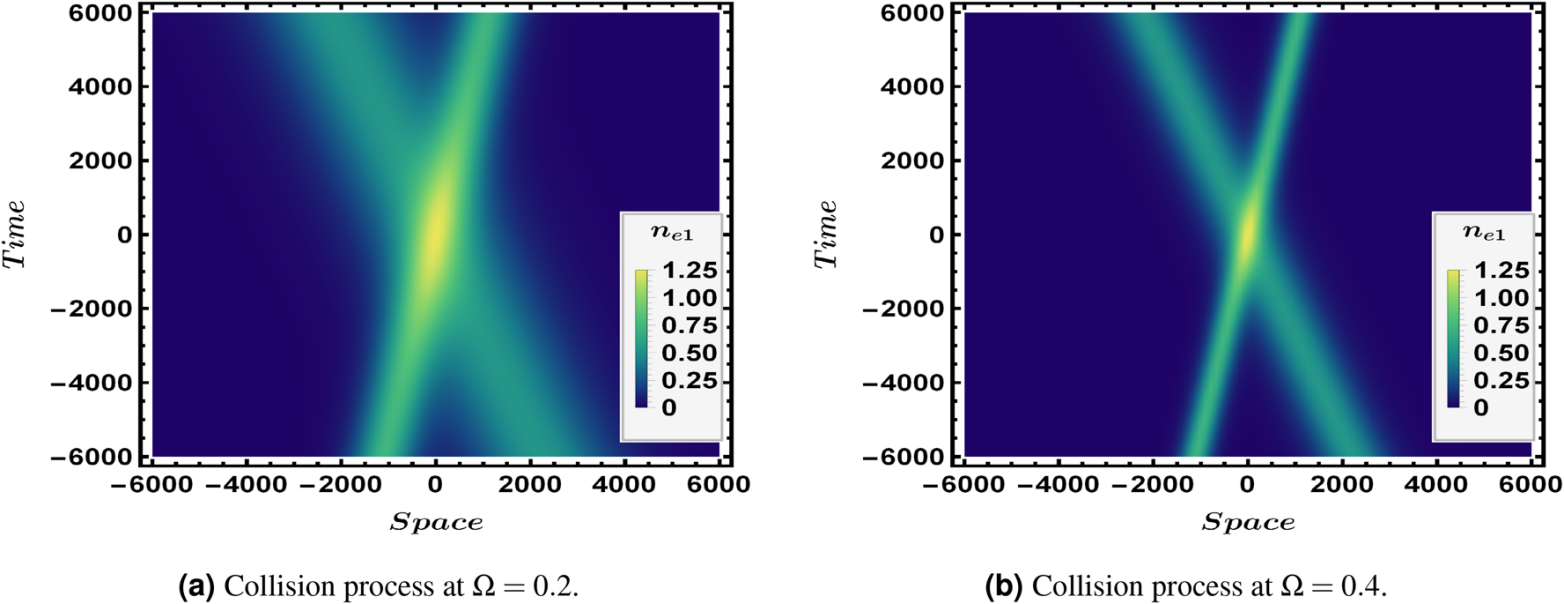
A density plot for the two solitons interaction at different values of $$\Omega $$ with $$\theta =48.2 {^{\circ }}$$, $$\epsilon =0.01$$, $$\phi {}_{1m}^{\eta }=0.3$$, $$\phi {}_{1m}^{\xi }=0.4$$ and $$n_{eo}=10^{35}$$$$m^{-3}$$.

The oblique collision at two different obliqueness angles is presented in Fig. 1 where the interaction at an angle $\theta =34.7 {^{\circ }}$ appears in Fig. 1a while Fig. 1b shows the collision at an angle $\theta =48.2 {^{\circ }}$ . Figure 2 illustrates the interaction (oblique collision) under the influence of different electron fluid number density $n_{eo}$, both Fig. 2a,b are plotted at $\epsilon =0.01$, $\Omega =0.2$ and $\theta =48.2 {^{\circ }}$. One can observe the direct effect of $n_{eo}$ on the soliton waves shape where in Fig. 2b the two solitons are much more sharper and apparently higher than the two interacting soliton waves in Fig. 2a. Figure 3 shows a density plot for the oblique collision of the two soliton solutions in Eqs. (34) and (35) where the impact of $\Omega$ is clear in the two panels of Fig. 3. Figure 3a is plotted for $\Omega =0.2$, while Fig. 3b is presented for $\Omega =0.4$ and all other conditions are the same in both which are $\theta =48.2 {^{\circ }}$, $\epsilon =0.01$ and $n_{eo}=10^{35}$. One can immediately deduce that the greater the value of $\Omega$, the narrower the width of the soliton becomes.

**Figure 4:**
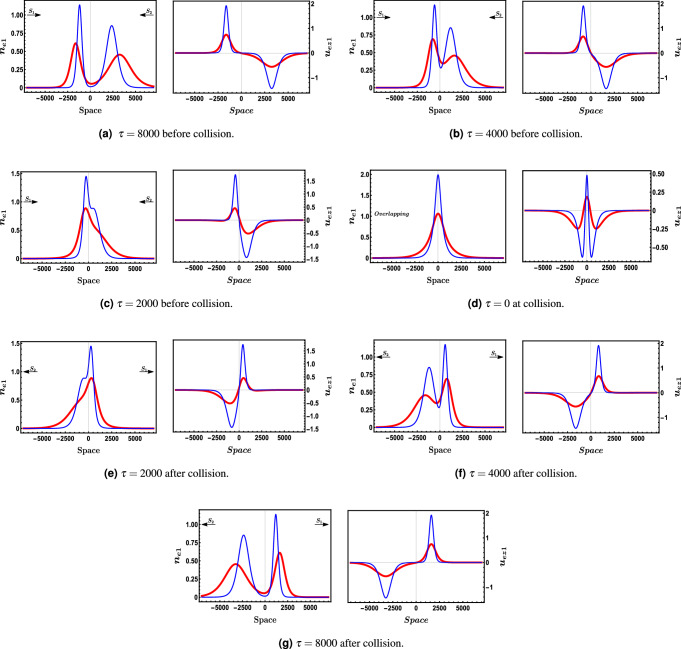
Depiction for the collision process,in a 2-D profile, of the two solitons via the first order perturbed quantities $$n_{e1}$$ and $$u_{ez1}$$ at an angle $$\theta =48.2 {^{\circ }}$$, $$\epsilon =0.01$$ and $$\Omega =0.2$$. Each panel has $$n_{e1}$$ (left) and $$u_{ez1}$$ (right). The blue curve is presented for $$\phi {}_{1m}^{\xi }=0.4$$ and $$n_{eo}=10^{36}$$$$m^{-3}$$ while the red thick curve is plotted for $$\phi {}_{1m}^{\eta }=0.4$$ and $$n_{eo}=10^{33}$$$$m^{-3}$$.

**Figure 5:**
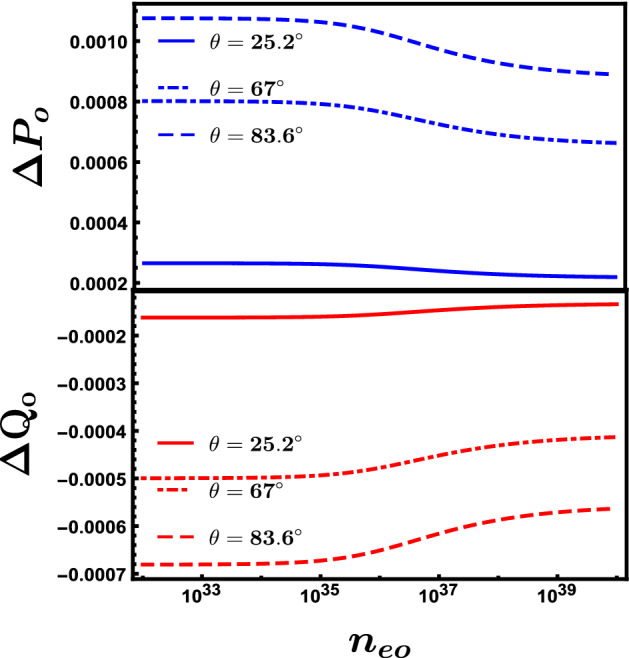
The demeanor of the $\triangle P_{o}$ and $\triangle Q_{o}$ against the electrons fluid number density $n_{eo}$ at different obliqueness angles $\theta$ where $\epsilon =0.01$, $\phi {}_{1m}^{\eta }=0.3$, $\phi {}_{1m}^{\xi }=0.4$ and $\Omega =0.2$.

Figure 4 represents a full interaction process for an oblique collision of the two solitons that are obtained as solutions for the KdV equations. In this figure also, Fig. 4, we introduced another aspect which is the influence of the equilibrium electrons fluid number density $n_{eo}$ on both the spacial occupying of the solitons and their shape, by introducing four solitons where each two of them are plotted at different $n_{eo}$. We presented the two blue solitons at $n_{eo}=10^{36}\,{\text {m}}^{-3}$ while the two red ones at $n_{eo}=10^{33}\,{\text {m}}^{-3}$. Seven time moments are introduced to illustrate the process prior to the interaction till collision and then separation. It is obvious that the effect of increasing the equilibrium electrons fluid number density $n_{eo}$ has a direct influence on the width of the soliton and spontaneously the height of the soliton of course where it gets sharper and higher with raising $n_{eo}$. The phase shifts $\triangle P_{o}$ and $\triangle Q_{o}$ due to the oblique collision are studied also. We find in general as illustrated in Fig. 5 that there is approximately an inverse proportionality between the phase shift $\triangle P_{o}$, $\triangle Q_{o}$ and $n_{eo}$ in the range of $n_{eo}\approx 10^{35}\,{\text {m}}^{-3}\,\text { to }\,10^{38}\,{\text {m}}^{-3}$, while for the range $10^{35}\,{\text {m}}^{-3}>n_{eo}>10^{38}\,{\text {m}}^{-3}$ the change in phase shifts is nearly unnoticed. Figure 5 also discusses the impact of the obliqueness angle $\theta$ on the phase shifts when they are plotted against the equilibrium electron fluid number density $n_{eo}$. One can notice that there is an increase in the phase shift when raising the angle $\theta$ at a fixed value of $n_{eo}$.

**Figure 6:**
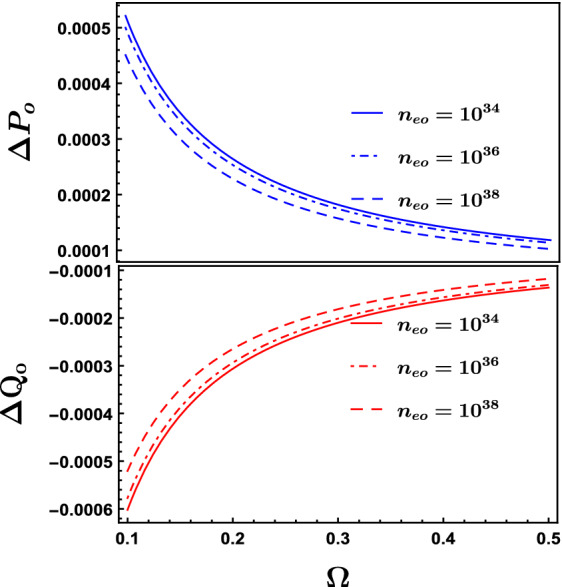
The demeanor of the $\triangle P_{o}$ and $\triangle Q_{o}$ against $\Omega$ at different equilibrium electrons fluid number density $n_{eo}$ where $\epsilon =0.01$, $\phi {}_{1m}^{\eta }=0.3$, $\phi {}_{1m}^{\xi }=0.4$ and $\theta =48.2 {^{\circ }}$.

Figure 6 clarifies the dependency of the phase shifts $\triangle P_{o}$ and $\triangle Q_{o}$ on $\Omega$ at different equilibrium electrons fluid number density $n_{eo}$, it is found that increasing $n_{eo}$ results in a decrease in the phase shifts in general. A notable inverse proportionality in Fig. 6 among the phase shifts $\triangle P_{o}$, $\triangle Q_{o}$ and $n_{eo}$, however with the higher values of $\Omega$ at $\Omega > 0.45$ and regardless the value of $n_{eo}$, the phase shifts tend to be constant against $\Omega$. Also Fig. 6 shows that at constant $\Omega$, the phase shifts get lower with raising the equilibrium electrons fluid number density $n_{eo}$ which agrees with the result obtained in Fig. 5.

**Figure 7:**
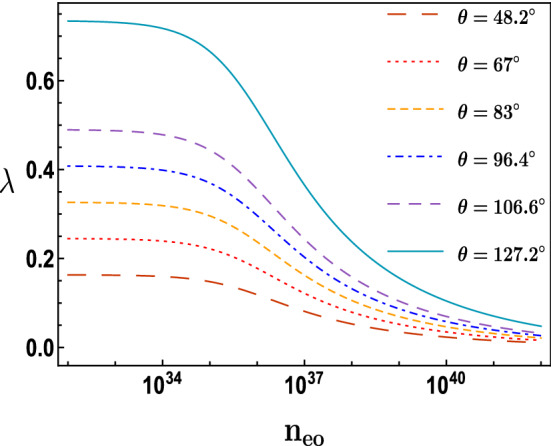
Dependency of the phase velocity $\lambda$ on the equilibrium electrons fluid number density $n_{eo}$ at different values of $\theta$ where $\epsilon =0.01$.

The impact of equilibrium electrons fluid number density $n_{eo}$ on the phase velocity is introduced in Fig. 7, in which it is found that an inverse proportionality between the phase velocity $\lambda$ and $n_{eo}$ which is similar to the range that was presented in Fig. 5 which is between $n_{eo}\approx 10^{35}\,{\text {m}}^{-3}\,\text { to }\,10^{38}\,{\text {m}}^{-3}$, on the other hand for the range $10^{35}\,{\text {m}}^{-3}>n_{eo}>10^{38}\,{\text {m}}^{-3}$ the phase velocity $\lambda$ tends to be nearly unchanged against $n_{eo}$ specially for higher values of obliqueness angle $\theta$. Figure 7 also shows that the higher the value of $\theta$, the greater the value of the phase velocity $\lambda$ specially for the range of the equilibrium electrons fluid number density $n_{eo} < 10^{35}\,{\text {m}}^{-3}$.

Our model would be a suitable theoretical model that can be applied to dense degenerate plasmas like intense laser–solid density plasma experiments^44,45^, astrophysical superdense bodies (e.g. the white dwarf core and neutron stars)^46,47^, the micro and nano scale quantum diodes^49,50^ and quantum free-electron lasers^58^ .

## Conclusion

As a summary, we have studied the oblique collision between two ion acoustic solitons waves (IASs) in a relativistic degenerate plasma that consists of degenerate electrons and nondegenerate cold ions. The extended Poincaré–Lighthill–Kuo (PLK) method has been used to study this interaction, getting two Korteweg deVries (KdV) wave equations describing two obliquely interacting solitons and obtaining the leading phase shifts due to the interaction. We studied mainly the effect of electrons fluid number density $n_{eo}$ on the interaction of the two solitons with each other, phase shifts and phase velocity. We find that the soliton wave gets more higher and sharper (narrower) in width as $n_{eo}$ increases. An inverse relationship between both the phase shifts $\triangle P_{o}$ and $\triangle Q_{o}$ and $n_{eo}$ in the range $n_{eo}\approx 10^{35}\,{\text {m}}^{-3}\,\text { to }\,10^{38}\,{\text {m}}^{-3}$ while for the range $10^{35}\,{\text {m}}^{-3}>n_{eo}>10^{38}\,{\text {m}}^{-3}$ there was approximately no phase shifts change against $n_{eo}$. An increase of the phase shifts at a constant value of $n_{eo}$ when raising the value of the obliqueness angle $\theta$ . The phase velocity showed inverse relationship with $n_{eo}$ in the range $n_{eo}\approx 10^{35}\,{\text {m}}^{-3}\,\text { to }\,10^{40}\,{\text {m}}^{-3}$, but for the range $10^{35}\,{\text {m}}^{-3}>n_{eo}>10^{40}\,{\text {m}}^{-3}$, there are nearly no change. Also we found that the phase velocity $\lambda$ increases with the raising of the obliqueness angle $\theta$ particularly for the range of the equilibrium electrons fluid number density $n_{eo} < 10^{35}\,{\text {m}}^{-3}$.
